# Bizarre parosteal osteochondromatous proliferation co-occurring with a metatarsal fatigue fracture: a case report

**DOI:** 10.1186/s12891-020-3168-x

**Published:** 2020-03-12

**Authors:** Ruoyu Yao, En Lin Goh, Zhaoxiang Fan, Xuequn Wu, Yanqi Feng

**Affiliations:** 1 Department of Orthopaedics and Traumatology, Longhua Hospital, Shanghai University of Traditional Chinese Medicine, Shanghai, 200032 China; 2 Oxford University Clinical Academic Graduate School, Medical Sciences Division, University of Oxford, Oxford, OX3 9DU UK

**Keywords:** Bone tumour, Bizarre parosteal osteochondromatous proliferation, Nora’s lesion, Osteochondroma, Fatigue fracture

## Abstract

**Background:**

Bizarre parosteal osteochondromatous proliferation (BPOP) is a relatively rare benign extraperiosteal osteochondroma-like proliferative lesion that shares similarities with malignant tumours in terms of morphology. The aetiology of BPOP has yet to be determined and there are no previous reports of BPOP associated with fracture.

**Case presentation:**

A 57-year-old woman presented with a one-month history of pain and swelling in her right foot, which were worsened by activity and improved with rest. Physical examination revealed a hard, non-mobile mass measuring 1.5 cm × 1.5 cm on the dorsal aspect of the third metatarsal of the right foot. There was overlying erythema and tenderness on palpation. Computed tomography (CT) of the right foot demonstrated a fracture of the neck of the third metatarsal, osteolysis at the fracture site and soft tissue swelling. Bone scintigraphy revealed increased tracer uptake suggesting abnormal bone metabolism at the neck of the third metatarsal. Surgical excision of the lesion was performed. Histopathology and immunohistochemistry confirmed the diagnosis of BPOP.

**Conclusion:**

BPOP is a rare benign lesion that is commonly misdiagnosed. Differential diagnosis is mainly achieved through imaging and histopathological assessment.

## Background

Bizzare parosteal osteochondromatous proliferation (BPOP) is a relatively rare benign extraperiosteal osteochondroma-like proliferative lesion that was first described by Nora et al. [1] in 1983, who reported 35 cases of this tumour affecting the hands and feet. This condition commonly affects the small bones of the hands and feet and rarely the long bones, vertebrae, skull and jaw. There is a peak incidence in adults between the second and third decade of life, with an equal preponderance for males and females [1]. BPOP typically presents as a painful, bony swelling that progressively increases in size over a period of weeks to months in the absence of trauma. This lesion grows rapidly and exhibits features on imaging and histopathological examination that are similar to malignant tumours including chondrosarcomas and osteosarcomas, leading to diagnostic uncertainty [1, 2]. The aetiology of BPOP has yet to be determined.

Due to the rarity of BPOP, there is limited evidence in the literature regarding the clinical features, investigation findings and management of this condition. We present a case of BPOP co-occurring with a metatarsal fatigue fracture. This case report has been presented in line with the Case Report (CARE) guidelines [3].

## Case presentation

**Fig. 1:**
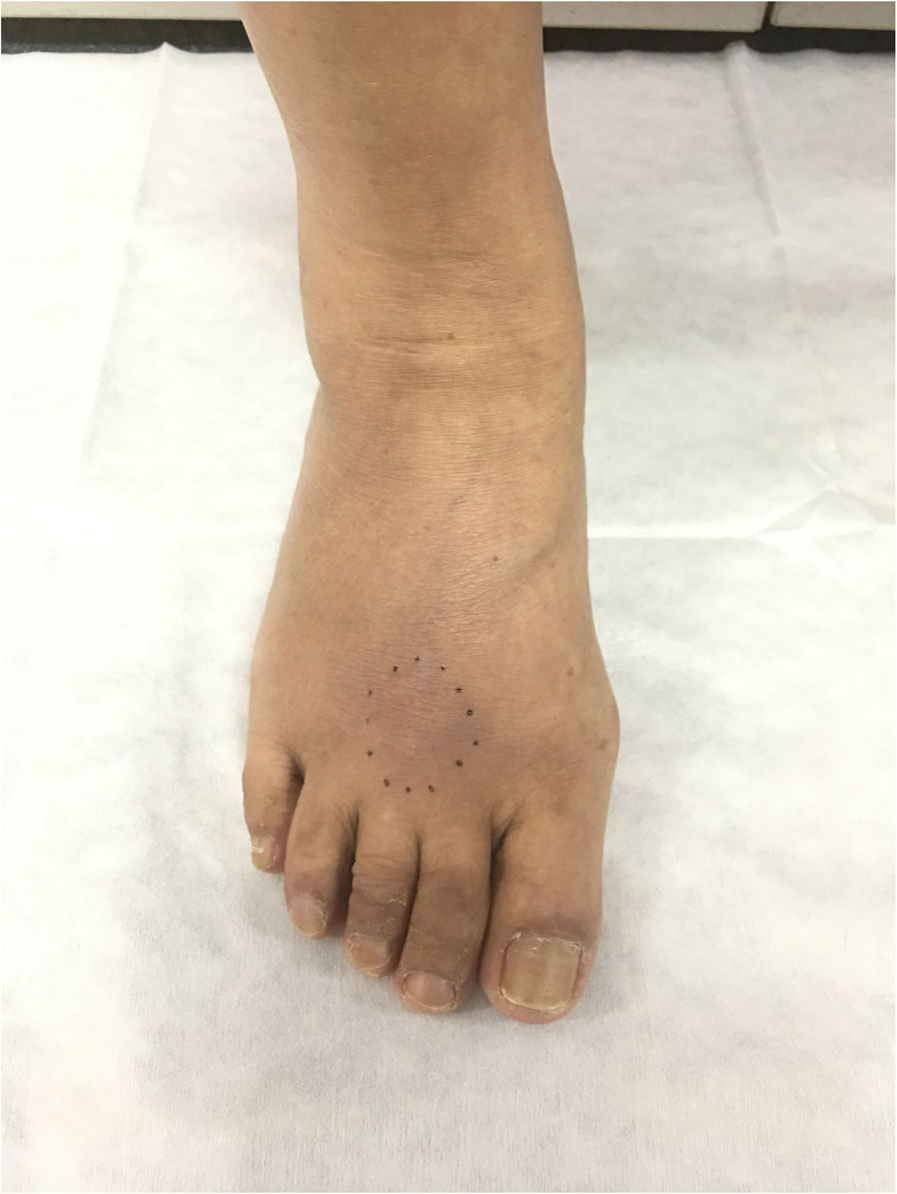
Picture of the affected foot, with the black dots highlighting the abnormal area

A 57-year-old woman presented with a one-month history of pain and swelling in her right foot, which were worsened by activity and improved with rest. She was reviewed at the onset of symptoms and diagnosed with cellulitis for which she was unsuccessfully treated with antibiotics. She remained physically active and independent of all activities of daily living with no physical restrictions. There was no history of preceding trauma. Physical examination revealed a hard, non-mobile mass measuring 1.5 cm × 1.5 cm on the dorsal aspect of the third metatarsal of the right foot (Fig. 1). There was overlying erythema and tenderness on palpation.

**Fig. 2:**
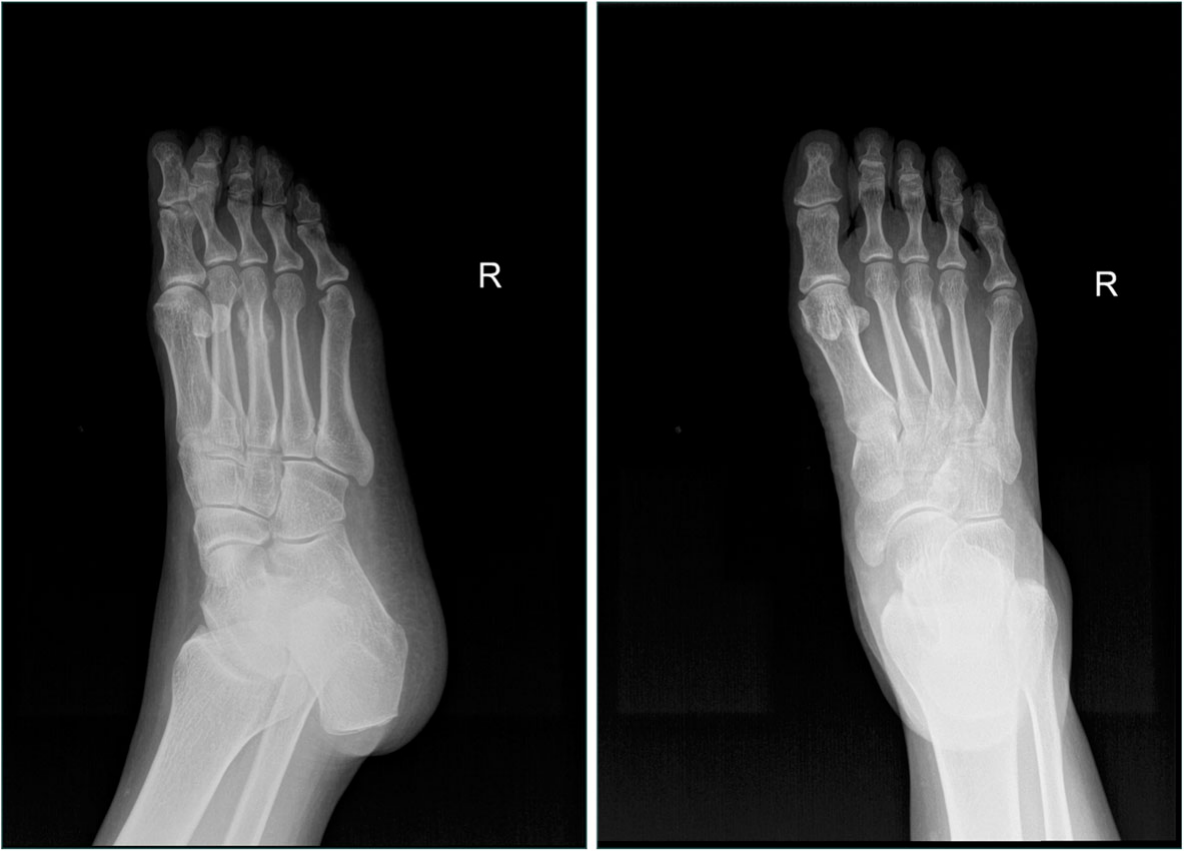
Plain X-rays of the right foot showing the lesion on the neck of the third metatarsal, with narrowing of the medullary cavity, with no visible fracture line

**Fig. 3:**
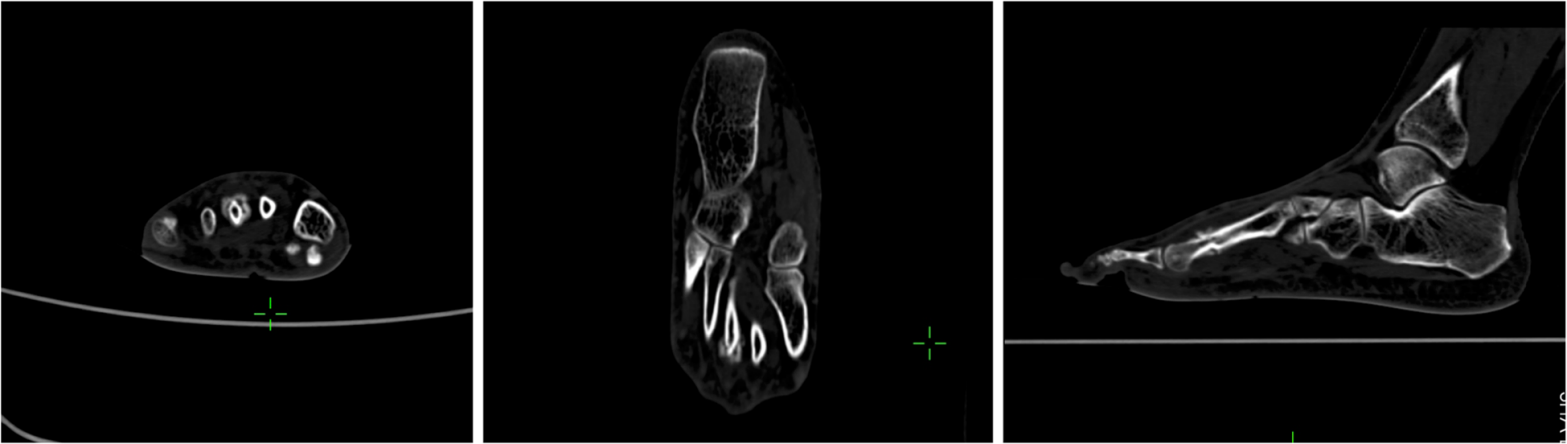
CT imaging of the right foot showing the continuity of the lesion with the cortex and interruption of bone continuity

**Fig. 4:**
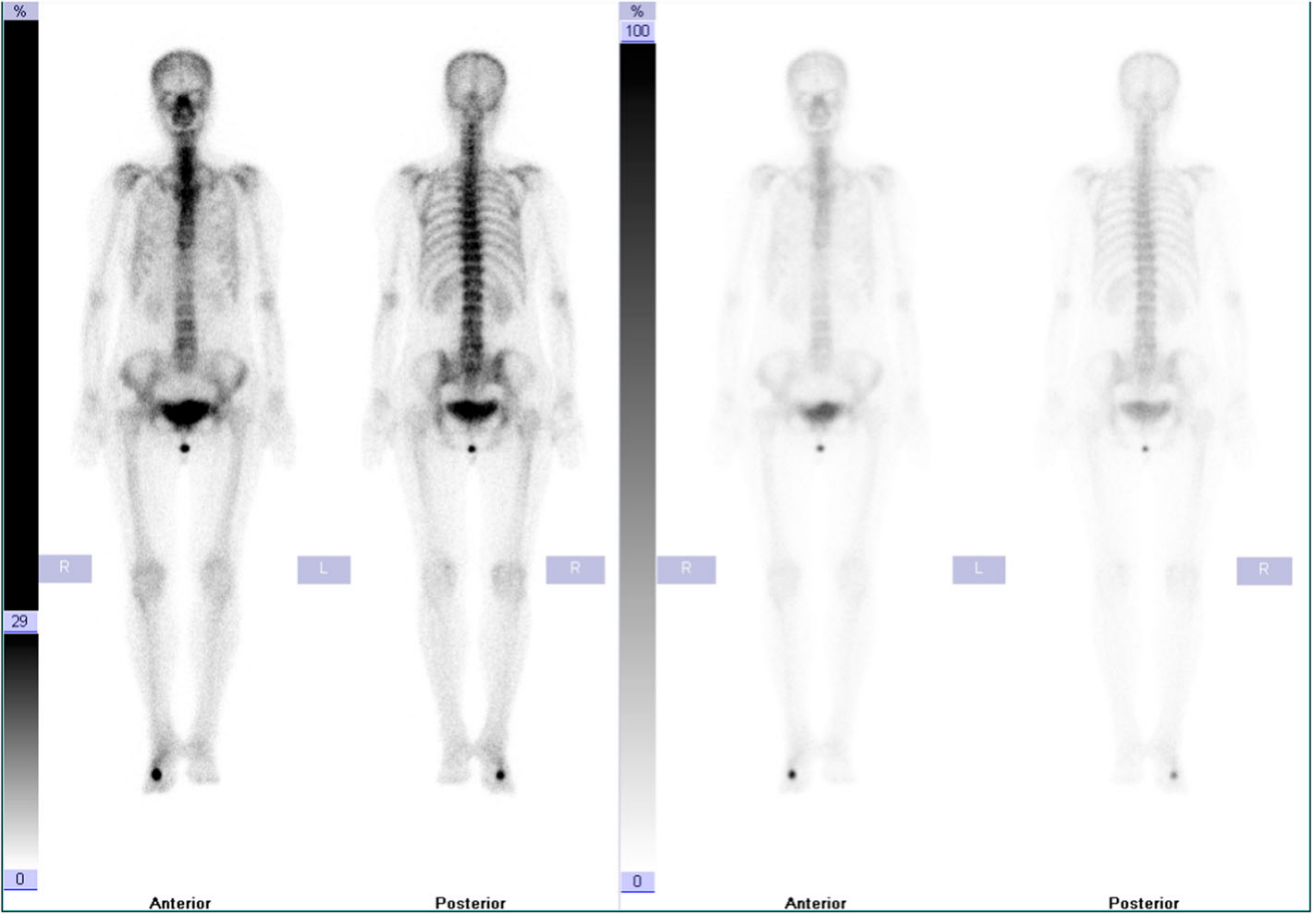
Bone scintigraphy showing increased tracer uptake in the neck of the third metatarsal on the right foot

All laboratory results were normal. Plain X-rays of the right foot showed an irregular 1.5 cm × 1.5 cm bony mass at the neck of the third metatarsal with narrowing of the medullary cavity (Fig. 2). Computed tomography (CT) of the right foot demonstrated a fracture of the neck of the third metatarsal, osteolysis at the fracture site and soft tissue swelling (Fig. 3). Bone scintigraphy showed increased tracer uptake suggesting abnormal bone metabolism at the neck of the third metatarsal (Fig. 4).

She underwent surgical excision of the lesion. An incision was made through the skin and subcutaneous tissue and the soft tissue was dissected to expose a hard, greyish-white, coarse, irregularly shaped osseous mass measuring about 1.5 cm × 1.5 cm × 1 cm, which was adherent to the neck of the third metatarsal. The mass was removed along the cortical surface and a transverse fracture line was found on the metatarsal neck. The fracture was stable and undisplaced.

**Fig. 5:**
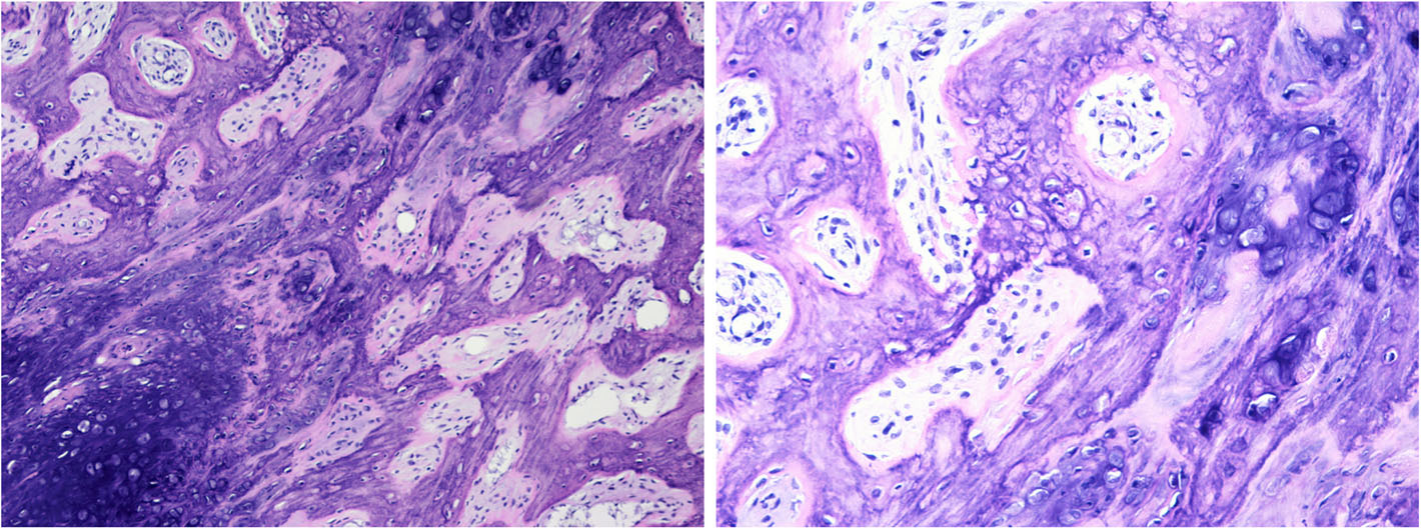
Under 100X magnification microscope observation, bone, cartilage, and fibrous tissue were mixed and irregularly distributed. Under 200X magnification observation, bone trabeculae were irregularly distributed and surrounded by osteoblasts (no atypia). Massive and patchy hyaline cartilage was observed, with the presence of the characteristic ‘Blue Bone’ and mildly atypical chondrocytes; Fibrous tissue, without any atypia, was located between bone trabeculae and cartilage

Histopathology revealed maturely differentiated bone, cartilage, and fibrous tissue. Fibrous composition was observed between trabecular bones. Some chondrocytes showed mildly atypical features indicating a diagnosis of the BPOP (Fig. 5). Immunohistochemistry: Ki-67 (+ 10%), SMA (+), S-100 (−), CD68 (scattered +), Bcl-2 (−), CD34 (−), CD19 (−), Caponin (+), Desmin (−), EMA (−).

Following surgery, plaster immobilisation was performed and removed after 6 weeks. Repeat X-rays after 6 weeks showed a healed third metatarsal with no signs of recurrence. She underwent physiotherapy and was followed up at regular monthly intervals for 1 year, with no post-operative sequelae.

## Discussion and conclusions

There were several key learning points from this case. A comprehensive assessment and work-up were performed, enabling prompt diagnosis and treatment of this condition. Given the rarity of this condition, the patient was followed up at regular intervals for 1 year, which provides longitudinal data on the clinical course of this condition. Nonetheless, the diagnosis of this condition remains challenging even with current imaging modalities and the long-term outcomes such as risk of recurrence are yet to be fully understood.

Yuen and colleagues proposed that this lesion occurs as part of reparative process following an inciting event, often related to trauma [4]. This was supported by Ly et al. who reported the development of this lesion in the upper limb following a traumatic event [5]. Similarly, a history of trauma was elucidated in 30% of the cases described by Meneses and colleagues [2]. It must be noted that the majority of patients do not report any significant history of trauma. A genetic component has been implicated, with recent studies suggesting that BPOP is associated with chromosomal translocations involving t(1;17)(q32;q21) and t(1;17)(q42;q23) [6, 7].

BPOP does not typically adhere to the cortex and has no continuity with the normal medulla. However, Rybak et al. reported four cases in which the lesion was continuous with the cortex and medulla [8]. Barrera-Ochoa et al. also reported two atypical BPOP cases with lesion and attached bone adhesion and marrow cavity invasion [9]. Pain and swelling were predominant symptoms and were associated with skin erythema, ulceration and gangrene. In the present case, the lesion was adherent to the cortex, thus masking the fracture line on the initial X-ray. Although CT imaging identified the fracture, the erosion of cortex and medullary cavity caused by the lesion was not displayed. Atypical BPOP can be difficult to distinguish from malignant lesions such as chondrosarcoma and osteosarcoma and a high degree of suspicion is required. Surgical resection is the mainstay of treatment and provides a definitive diagnosis based on histopathology. Berber et al. recommend surgical resection of the lesion and preservation of surrounding tissues to reduce post-operative complications [10]. Long-term follow-up is necessary as the recurrence rate can be as high as 55% [11].

In conclusion, BPOP is a rare benign lesion that is commonly misdiagnosed. Differential diagnosis is mainly achieved through imaging and histopathological assessment. The importance of clinical history taking and pathological examinations must be emphasised. Further research is necessary to fully elucidate the pathogenesis of this disorder.

## Data Availability

Not applicable.

## Abbreviations

BPOP: Bizzare parosteal osteochondromatous proliferation
CT: Computed tomography

## References

[1] Nora FE, Dahlin DC, Beabout JW (1983). Bizarre parosteal osteochondromatous proliferations of the hands and feet. Am J Surg Pathol.

[2] Meneses MF, Unni KK, Swee RG (1993). Bizarre parosteal osteochondromatous proliferation of bone (Nora’s lesion). Am J Surg Pathol.

[3] Gagnier JJ, Kienle G, Altman DG, Moher D, Sox H, Riley D (2013). The CARE guidelines: consensus-based clinical case reporting guideline development. J Med Case Rep.

[4] Yuen M, Friedman L, Orr W, Cockshott WP (1992). Proliferative periosteal processes of phalanges: a unitary hypothesis. Skelet Radiol.

[5] Ly JQ, Bui-Mansfield LT, Taylor DC (2004). Radiologic demonstration of temporal development of bizarre parosteal osteochondromatous proliferation. Clin Imaging.

[6] Nilsson M, Domanski HA, Mertens F, Mandahl N (2004). Molecular cytogenetic characterization of recurrent translocation breakpoints in bizarre parosteal osteochondromatous proliferation (Nora’s lesion). Hum Pathol.

[7] Endo M, Hasegawa T, Tashiro T, Yamaguchi U, Morimoto Y, Nakatani F (2005). Bizarre parosteal osteochondromatous proliferation with at (1; 17) translocation. Virchows Arch.

[8] Rybak LD, Abramovici L, Kenan S, Posner MA, Bonar F, Steiner GC (2007). Cortico-medullary continuity in bizarre parosteal osteochondromatous proliferation mimicking osteochondroma on imaging. Skelet Radiol.

[9] Barrera-Ochoa S, Lluch A, Gargallo-Margarit A, Pérez M, Vélez R. Bizarre Parosteal osteochondromatous proliferation (Nora’s lesion) of the hand: a report of two atypical cases. Case Rep Med. 2012;2012:453560. 10.1155/2012/453560 PMC3541688 23326274

[10] Berber O, Dawson-Bowling S, Jalgaonkar A, Miles J, Pollock RC, Skinner JA (2011). Bizarre parosteal osteochondromatous proliferation of bone: clinical management of a series of 22 cases. J Bone Joint Surg Br.

[11] Singh R, Jain M, Siwach R, Rohilla RK, Kaur K (2010). Unusual presentation of bizarre parosteal osteochondromatous lesion of the second toe (Nora’s lesion). Foot Ankle Spec.

